# Acute flaccid paralysis surveillance indicators in the Democratic Republic of Congo during 2008-2014

**DOI:** 10.11604/pamj.2016.24.154.8747

**Published:** 2016-06-22

**Authors:** Hugo Kavunga Membo, Aaron Mweene, Serge Alain Sadeuh-Mba, Justin Masumu, Riziki Yogolelo, Norbert Ngendabanyikwa, Eddy Sokolua, Fred Sagamiko, Edgar Simulundu, Steve Ahuka, Jean Jacques Muyembe

**Affiliations:** 1 Institut National de Recherche Biomédicale (INRB), P.O Box 1197 Kinshasa 1, Kinshasa, Democratic Republic of Congo; 2 Department of Disease Control, School of Veterinary Medicine, University of Zambia, P.O Box 32379, Lusaka, Zambia; 3 Service de Virologie, Centre Pasteur du Cameroun (CPC), rue Henri Dunant P.O Box 1274, Yaoundé, Cameroun; 4 World Heath Organization (WHO), Central African Inter-country Bureau, Libreville, Gabon

**Keywords:** Poliomyelitis, surveillance, paralysis, poliovirus, indicators, Democratic Republic of Congo

## Abstract

**Introduction:**

The last wild poliovirus (WPV) case in Africa was reported in July 2014, thus underscoring the tremendous progress towards polio eradication worldwide. This study aimed to analyze the results of a seven-year surveillance of Acute Flaccid Paralysis (AFP) in the Democratic Republic of Congo (DRC) and to identify potential gaps that need to be addressed.

**Methods:**

Epidemiological and virological data obtained from AFP surveillance among AFP cases less than 15 years from January 2008 to December 2014 in DRC were retrospectively considered and analyzed in this study.

**Results:**

Of the 13,749 AFP cases investigated, 58.9% received at least three doses of oral polio vaccine (OPV), 7.3% never received OPV, while the status of 18.3% was unknown. Analysis of surveillance performances showed that all, but two, indicators were below the required WHO-specified targets. Non-polio enterovirus (NPEV) isolation rate was consistently below the minimum requirement at ≥10% and the proportions of stool specimens that reached the laboratory within 72 hours of being sent were always below 15% (WHO target is ≥80%). Virus isolation and differentiation showed that 1.5% of AFP cases were infected by WPVs, 5.5% by Sabin strains, 0.5% by vaccine-derived polioviruses (VDPVs) and 7.2% by NPEVs.

**Conclusion:**

Our findings indicate that additional efforts are needed to address the timeliness of adequate stool specimens’ arrival to the laboratory. It remains essential to maintain high polio vaccine coverage and high AFP surveillance standards to ensure rapid detection and containment of either WPV importation or VDPV re-emergence in DRC.

## Introduction

Poliomyelitis is a highly infectious disease caused by the polioviruses (PVs) belonging to the species *Enterovirus C*, genus *Enterovirus* and family *Picornaviridae* [1]. Like most other Enteroviruses, PVs are transmitted by the feco-oral route. Poliomyelitis can affect individuals of any age, but primarily involves children aged less than five years. In up to 1% of those infected the virus invades the central nervous system leading to muscle weakness and irreversible paralysis (usually in the lower limbs), often progressing to breathing difficulties, and death [2, 3]. In 1988, the World Health Organization (WHO) adopted the Global Polio Eradication Initiative (GPEI), aimed at eradicating polio by the year 2000 [4]. The strategies of the GPEI include conducting surveillance for Acute Flaccid Paralysis (AFP) to determine the wild or vaccine-derived origin of PVs isolates, achieving high rate of routine polio immunization coverage of the population and carrying out supplemental immunization activities (SIA) in response to polio outbreaks. Two vaccines efficiently prevent the onset of poliomyelitis. Inactivated Polio Vaccine (IPV) is very safe and induces a strong and protective general antibody response while Oral Polio Vaccine (OPV), used in mass vaccination campaigns in endemic regions, is made up of live attenuated viruses (Sabin strains of serotypes 1, 2 and 3) that are able to multiply to high titers in the gastrointestinal tract, thus inducing strong mucosal immunity to PVs [5]. The major disadvantage of OPV is that vaccine strains can replicate and mutate during inter human circulation in under immunized populations, thus leading to the emergence of neurovirulent vaccine derived PVs (VDPVs) [5, 6].

Although polio outbreaks continue to occur, the poliomyelitis incidence has drastically decreased by > 99%: from an estimated 350,000 cases in 1988 to 359 cases in 2014 [7]. The number of polio-endemic countries has also decreased from 125 countries in 1988, to only two countries in 2014 (Pakistan and Afghanistan) [7].

AFP is defined as acute or sudden onset of weakness or paralysis of a limb characterized as flaccid (reduced tone) in a child < 15 years of age [8]. AFP surveillance consists of the detection and investigation of flaccid paralysis of new onset in children less than 15 years or any other suspected poliomyelitis case in a person of any age. It has been adopted globally as an essential strategy for monitoring the progress of the polio eradication initiative [9]. Hence, AFP surveillance remains the “gold standard” to identify areas and populations who are at high risk, to assess polio status of a given country, to reveal the need of SIA and to certify the absence of wild PV (WPV) circulation in countries that are no longer reporting poliomyelitis cases [10].

In the Democratic Republic of Congo (DRC), the Expanded Program of Immunization (EPI) was introduced in 1978 with childhood vaccination schedule for polio including four doses of live-attenuated OPV at birth, 6, 10 and 14 months of age. The Polio Eradication Program led by the DRC's EPI started in 1996 and its efforts have shown how much polio vaccine contributed to the decrease of poliomyelitis cases throughout the country in such a way that by 2001, DRC was no longer polio endemic. However, WPV transmission was reestablished in 2006 continuing until the onset of the last case on December 20, 2011 [11]. In the other hand, circulating VDPVs were isolated from 2004 to 2012; with a large VDPVs associated poliomyelitis outbreak that affected the Kasai Oriental and Maniema provinces from 2008 to 2012 [12]. Moreover, DRC as any other country remains at risk of WPVs re-importation from the remaining polio-endemic reservoirs [13, 14].

The Institut National de Recherche Biomédicale (INRB), located at the capital city Kinshasa, is the WHO-accredited National Reference Poliomyelitis Laboratory (NRPL) in DRC. INRB has routinely registered epidemiological and virological data from AFP surveillance since 1997. Regular analyses of data generated from an AFP surveillance system are of great interest for the identification of potential problems to be addressed in order to guarantee timely detection of WPV re-importation or VDPV re-emergence. WHO developed a set of performance indicators to ensure that AFP surveillance is conducted with the required standards, maintained and operated uniformly [8].

This study describes the results of the surveillance of AFP in DRC from January 2008 to December 2014 and identifies the gaps that need to be addressed in order to provide the highest level of confidence in the control and prevention of PV infection in DRC.

## Methods

### Study area and design

The DRC is located in sub-Saharan Africa, bordered by Angola, the South Atlantic Ocean, the Republic of Congo, the Central African Republic, South Sudan, Uganda, Rwanda, Burundi, Tanzania across Lake Tanganyika, and Zambia. The country is divided into eleven administrative provinces with 65 districts and 516 health zones [15]. The health system is organized according to national, provincial, district and health zone levels. According to the 2015 census, DRC is estimated to have a total population of about 70 million people with children under the age of 15 years being about 43 million [15]. Here, a retrospective descriptive study was conducted using AFP surveillance data routinely collected from January 2008 to December 2014 by the INRB at Kinshasa. All AFP cases reported nationwide during the studied period were considered for this study.

### Organisation of AFP surveillance in DRC

Data for AFP surveillance is routinely registered in the weekly and monthly reporting system from the Health Zones and districts of all provinces of DRC. An AFP case is defined as any child < 15 years who develops acute onset of focal weakness or paralysis characterized as flaccid (including Guillain Barre Syndrome), without any other obvious cause [9, 10]. When a patient meeting the AFP case definition is presented at a health facility, the health care practitioners conduct comprehensive investigations to find out if PV is the cause of the paralysis. Investigations include recording of detailed history, conducting a systematic examination, and collecting two stool specimens, 24 to 48 hours apart, within 14 days of onset of symptoms. The case investigation form is filled with information on demographic, clinical history, vaccination history and dates of stool specimen collection to accompany the stool specimens sent to the WHO-accredited NRPL at the INRB for virological analysis. Virological analyses include virus isolation on Human rhabdomyosarcoma (RD) and murine L20B (a derivative of murine L cells expressing the PV human receptor) cell cultures and identification of the resulting isolates by intratypic differentiation (ITD) with a real time reverse transcriptase polymerase chain reaction (RT-PCR) assay [16–18]. Suspected PV and VDPVs strains are further characterized by the sequencing of the VP1-capsid coding gene [19].

### WHO Accredited National Reference Polio Laboratory

The WHO-accredited NRPL is located at the INRB in Kinshasa, the capital city of DRC. The NRPL follows standardized procedures for i) the isolation of PV and non polio enteroviruses (NPEVs) using RD and L20B cell cultures, ii) the PV identification to confirm WPV cases, iii) the differentiation of the three PV serotypes (1–3), WPVs, Sabin-like PVs and VDPVs by ITD and iv) the shipment of the suspected WPV and VDPV isolates to a WHO Specialized Polio Laboratory where sequencing of VP1 gene is performed. The timeliness, accuracy and quality of the NRPL are assessed through proficiency testing for laboratory techniques and annual onsite accreditation review [20].

### Indicators of AFP surveillance performance

WHO has established some minimum performance indicators that should be used to ensure that the quality of AFP surveillance is achieved and maintained. In this study, we evaluated the performance of the AFP surveillance system using the following WHO-specified indicators:

**Number of non-polio AFP cases per 100 000 population aged <15 years:** This is an indicator of the sensitivity of the AFP surveillance system. The system should be able to detect at least two AFP cases per 100,000 children below the age of 15 years. In the specific case of DRC, with an estimated 43,000,000 less than 15 years, 860 AFP cases a year is the minimum target.

**Percentage of AFP cases with two adequate stool specimens:** Adequate stools are defined as two stool specimens collected from an AFP patient 24-48 hours apart and within 14 days of onset of symptoms. At least 80% of all AFP cases should have adequate stool specimens.

**Condition of stool on arrival at laboratory:** At least 80% of the stool specimens should arrive at the WHO accredited laboratory in “good condition”. A stool specimen is said to have arrived in good condition if it was transported under reverse cold chain conditions (with ice packs and a temperature indicator) and was received by the WHO accredited polio isolation laboratory in sufficient quantity (at least 8 grams) and with appropriate documentation.

**Percentage of AFP cases investigated within 48 hours of being notified <48 hours:** At least 80% of AFP cases should be investigated within 48 hours of being notified.

**Percentage of stool specimens received in the laboratory within 3 days of collection:** At least 80% of stool specimens collected from AFP cases should arrive at a WHO accredited polio isolation laboratory within 72 hours of being sent.

**Percentage of specimen results sent from national laboratory within 14 days of receipt of the specimen in the laboratory:** At least 80% of specimen results should be sent from the polio isolation laboratory within 14 days of specimen receipt by the laboratory while ITD results should be reported within 7 days of receipt

**Percentage of stool specimens from which an NPEV is isolated:** At least 10% of stool specimens submitted to the laboratory should have an NPEV isolated. This is an indicator of the quality of the reverse cold chain and how well the laboratory is able to perform routine isolation of enterovirus.

**Percentage of AFP cases followed up at least 60 days from the onset:** At least 80% of AFP cases requiring a follow-up examination should be reviewed at 60 days after the onset of paralysis by a national expert committee, to verify the presence of residual paralysis or weakness.

## Results

**Figure 1:**
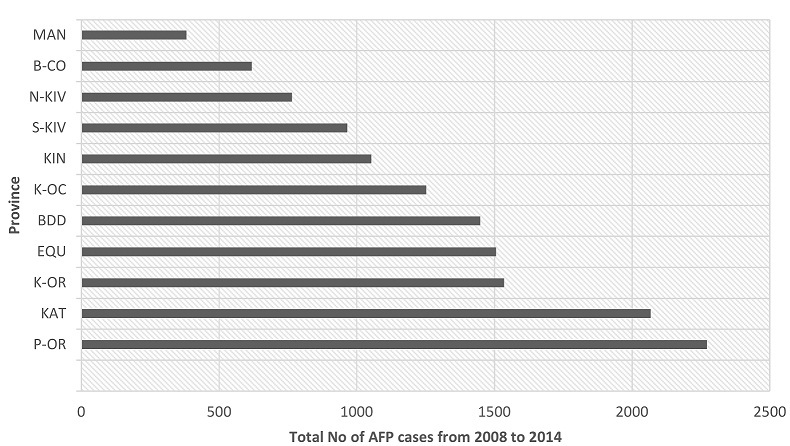
Number of AFP cases reported by Province in the DR Congo, 2008-2014. MAN = Maniema, B-CO = Bas Congo, N-KIV= Nord Kivu, S-KIV = Sud Kivu, KIN = Kinshasa, K-OC = Kasai Occidental, BDD = Bandundu, EQU = Equateur, K-OR= Kasai Oriental, KAT = Katanga, P-OR = Province Orientale

**Figure 2:**
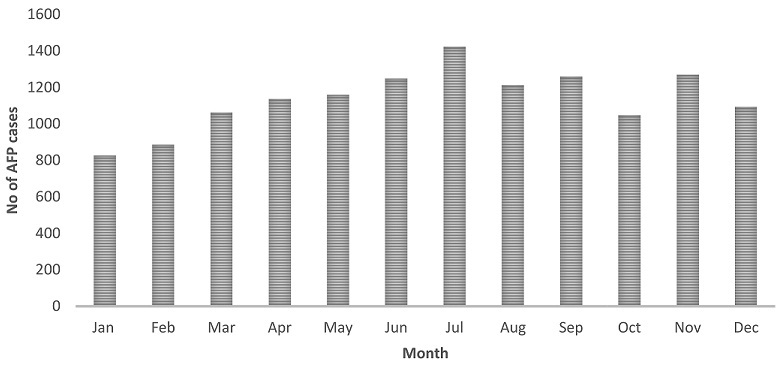
Number of AFP cases reported by month from 2008-2014. Jan = January, Feb= February, Mar = March, Apr = April, May= May, Jun= June, Jul = July, Aug = August, Sep= September, Oct = October, Nov= November; Dec= December

**Figure 3:**
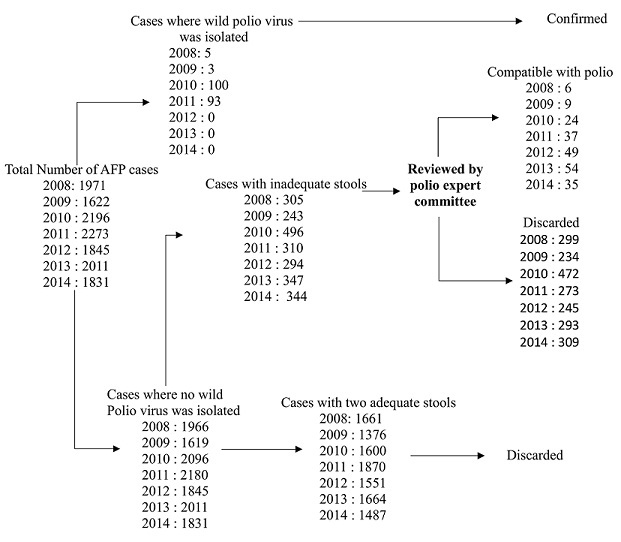
Flow chart showing the virological classification of AFP cases reported in DRC between 2008 and 2014

From January 2008 to December 2014, a total of 13,749 cases of AFP were reported nationwide by the surveillance system in DRC. Stool specimens originated from all eleven provinces of the country with the minority of them coming from the MANIEMA province (2.8%) and the majority from Province Orientale (16.5%) (Figure 1). The lowest numbers of cumulated cases were recorded in January and February with 6% and 6.4% respectively, while the highest number of cumulated cases was observed in July (10.3%) (Figure 2). Among the 13,749 AFP cases analysed, the largest proportion (85.2%) involved children under the age of 5 years and about 55% were from males (Table 1). The immunization status records showed that 15.5% of the children had received less than three doses of OPV, 58.9% had received at least three doses of OPV, 7.3% had never received any oral polio vaccine while the vaccination status of 18.3% of the children was unknown. As shown in Table 1 94.7% of the AFP cases had sudden onset of paralysis, 82.7% had fever at the onset of paralysis and less than half (42.1%) had asymmetric paralysis. Of all 13,749 AFP cases analyzed, 201 (1.5%) were classified as WPV cases, 768 (5.5%) as Sabin cases, 68 (0.5%) as circulating VDPV cases and 998 (7.2%) as NPEV cases. All recorded AFP cases could be classified as depicted in the virological classification flowchart presented in Figure 3. The National Expert Committee classified overall 214 (9.1%) cases with inadequate stool specimens as polio compatible while the remaining 2125 (90.9%) AFP cases with inadequate stool specimens were discarded (Figure 3).

**Table 1:** Proportions of AFP by year, age, sex, OPV doses and clinical symptoms in DRC from 2008 to 2014

Variable	Year						
Age	2008	2009	2010	2011	2012	2013	2014
< 5	88.5	90.3	89.9	83.8	84.3	80.1	80
5-10	7.8	7	5.9	7.0	7.7	11.2	11.8
>11	3.6	2.5	4.1	9.1	7.9	8.6	8.1
**Sex**							
Male	55.9	52.7	54.2	57.2	56	54.3	54.5
Female	43.8	47.2	45.6	42.7	43.9	45.6	44.6
**OPV doses**							
Zero dose (Not vaccinated)	7.8	12	11.8	7.7	5.6	3.2	2.8
< 3 doses	16.9	17.1	17.6	16.7	14.6	13	12
≥ 3 doses	61	59	55.2	53.7	60.6	62	62.4
Unknown	14.3	11.9	15.4	21.9	19.2	21.8	22.8
**Clinical symptoms**							
Sudden onset of paralysis	96.1	97.0	94.4	94.5	95.5	93.7	92.2
Fever	82.8	83.2	83.2	84.9	83.7	80.1	80.6
Asymmetrical	46.7	45	41.7	41	41.7	37.8	41.5

The circulation of WPV has been stopped over the country since 2011 [13]. All WPV cases obtained during the study period were genetically characterized and results from this study showed that the most frequently serotype was WPV type 1 while WPV type 2 were not detected (data not shown). As expected, no WPV type 2 was isolated as previous reported worldwide [21]. WPV type 3 cases were recorded only in 2008 and 2009 (Table 2). However, circulating VDPVs type 2 were repeatedly isolated during a large poliomyelitis outbreaks from 2008 to 2012 while an ambiguous VDPV type 2 (without evidence of inter human transmission) was found in 2014 (Table 2).

**Table 2:** Case number of AFP patient's under-15-year-olds, WPV, Sabin, NPEV, and VDPV isolates collected, by year in DRC from 2008 to 2014

Cases a	Year							Total n (%) c
	2008	2009	2010	2011	2012	2013	2014	
**Wild Polioviruses**								
wild PV type 1	4	0	100	93	0	0	0	197 (1.43)
wild PV type 2	0	0	0	0	0	0	0	0 (0.0)
wild PV type 3	1	3	0	0	0	0	0	4 (0.03)
Total wild Poliovirus	**5**	**3**	**100**	**93**	**0**	**0**	**0**	**201 (1.5)**
**Vaccine-derived Polioviruses**								
VDPV type 1	0	0	0	0	0	0	0	0 (0.0)
VDPV type 2 b	13	5	19	13	17	0	1	68 (0.49)
VDPV type 3	0	0	0	0	0	0	0	0 (0.0)
Total Vaccine-derived Polioviruses	**13**	**5**	**19**	**13**	**17**	**0**	**1**	**68 (0.49)**
Total Oral Polio Vaccine strains	**72**	**88**	**121**	**127**	**131**	**124**	**105**	**768 (5.58)**
Total Non Polio Enteroviruses	**91**	**91**	**206**	**223**	**154**	**121**	**112**	**998 (7.2)**
Total positive AFP cases	181	187	446	456	302	245	218	2035 (14.80)
Total negative AFP cases	1790	1435	1750	1817	1543	1766	1613	11714 (85.19)
Total all AFP cases	**1971**	**1622**	**2196**	**2273**	**1845**	**2011**	**1831**	**13749 (100)**

a PV, poliovirus; VDPV, vaccine-derived poliovirus; AFP, Acute Flaccid Paralysis;

b the VDPV case found in 2014 was unlinked to the 2008-2012 outbreak and no evidence of inter human transmission was found

c percentage among all AFP cases

The WHO defined the target of at least two AFP cases per 100 000 in children under 15 years as an indicator for measuring the sensitivity of AFP surveillance. This target was consistently achieved from 2008 to 2014. The proportion of AFP cases with 2 stool specimens collected within 14 days of onset of paralysis, during the investigation of AFP cases, was maintained above the WHO-specified national target of at least 80% except in 2010 where it was at 73% (Table 3). Two important indicators of AFP surveillance performance were consistently below the minimum standard throughout the study period. Despite the fact that most specimens arrived at the laboratory in good conditions, the proportion of stool specimens arriving at national level within three days of being sent did not reach 15% which is far below the acceptable minimum of 80% (Table 3). Moreover, the annualized non polio AFP rates that reveal the sensitivity of the system were consistently < 10% throughout the study period (Table 3).

**Table 3:** AFP surveillance performance indicators in DRC from 2008 to 2014

Parameter	Year								Average
	target	2008	2009	2010	2011	2012	2013	2014	
Annualized non-polio AFP rate 2/100,000 < 15 yrs population	≥2	5.8	4.5	5.8	5.4	4.4	4.2	3.9	4.8
Proportion of AFP cases investigated (out of the total notified)	≥80%	100%	100%	100%	100%	100%	100%	100%	100%
Proportion of AFP cases with 2 stool specimens collected within 14 days of onset of paralysis	≥80%	95%	85%	73%	82%	83%	83%	81%	83.1%
Proportion of stool specimens arriving at national level within 3 days of being sent (%)	≥80%	12%	9%	12%	13%	6%	7%	10%	9.8%
Proportion of stool specimens arriving at national lab in good condition	≥80%	93%	95%	88%	95%	97%	95%	95%	94%
Proportion of stool specimens for which lab results were sent within 14 days of receipt at the lab (%)	≥80%	95%	92%	85%	80%	96%	94%	93%	90.7%
Proportion of stool specimens from which non-polio enterovirus was isolated (%)	≥10%	4.6%	5.6%	9.3%	9.8%	8.3%	6%	6.1%	7.1%

## Discussion

The present study reports the incidence of AFP over seven-year (2008-2014) period in DRC. The performance of AFP surveillance over these years was also evaluated. The age distribution of AFP cases confirmed that low age was a risk factor as previously shown [22]. Accordingly, our results showed that 85.2% of AFP cases occurred in children below five years of age. Similarly, previous studies reported a frequency of 76.3% and 90% among this age group in Ghana [23] and in India [24], respectively. This indicates that though poliomyelitis can affect any age group, children under the age of five years predominantly get affected [10, 25]. Our findings indicate that the majority (55%) of AFP cases involved males. As described in 2008, by D'Errico et al. [26], the frequency of AFP was found to be consistently higher over the period of study among males than females with the mean incidence rate of 55% and 44.7% for boys and girls, respectively. This can be explained by the fact that there are sex differences in the susceptibility to infectious agents. Generally, males are more prone to develop an infectious disease than females and this may be probably related to genetic differences in immunologic responses [26].

This study revealed vaccination coverage with at least three OPV doses at 58.9% among AFP cases, which is lower compared to coverage rate found in Ikwa bom state in Nigeria (98.3%) [27]. This phenomenon may be explained by the fact that some parents failed to complete routine immunization (only one or two were given). Other parents had forgotten the number of OPV their children had taken since mass immunization campaigns are not systematically recorded. The apparent absence of WPVs and VDPVs circulation since 2012 is inconsistent with high vaccine failure among children. Hence, the actual coverage of the populations was likely above the estimates from field records especially after immunization responses to the 2008-2012 VDPV related poliomyelitis outbreaks. Accordingly, national polio vaccine coverage rate was estimated to have risen from 64% at the beginning in 2008 to at least 76% from 2009 to 2013 [28]. We found higher proportion of children with sudden onset of paralysis (94.7%) and 82.7% of cases had fever at onset which was higher than that reported in 2013 by Sevencan *et al* [29], who found 15% of AFP cases with prodromal fever in Turkey. Regarding the performance of the AFP surveillance in DRC, the findings of the present study mirrors those found previously, from 1997 to 2001 in DRC [30] in that AFP surveillance indicators were maintained above the WHO specified targets. The success of an AFP surveillance system does not only depend on the detection of AFP cases, but also hinges on the investigation and reporting of the cases. Results from DRC's AFP surveillance showed that the country′s overall proportion of adequate stool specimens, during the investigation of AFP cases, was maintained above the WHO-specified national target of at least 80% from 2008 to 2014. However, the average NPEV isolation rate was consistently below the minimum requirement throughout the study period (Table 2). These NPEV isolation rates were lower than the 12% and 17.6% reported in Ghana [23] and Iraq [31], respectively. This could be explained by the fact that many NPEV strains belonging to the *Enterovirus C* species cannot be isolated on RD cells. Previous studies showed that the introduction of HEp-2c cell line in the isolation algorithm significantly increases the NPEV isolation rate in tropical settings [32, 33]. Moreover, the timeliness of transportation of the stool specimens is critical for the surveillance system as enteroviruses should survive until the time of analysis for the laboratory to be able to isolate them [34]. According to the WHO-specified national targets for AFP surveillance, at least 80% of stool specimens must arrive at the laboratory within 72 hours of collection [35–37]. Results from this study reveal that this target remained out of reach throughout the study period. The proportion of specimens that arrived at the laboratory within 72 hours was consistently less than 15% whereas it was expected to be at?80%. This low performance for specimen's transportation to the laboratory may be due to the big size of the country as well as the lack of good roads in remote areas. This issue could be overcome by setting up sub-national polio surveillance laboratories besides the NRPL in Kinshasa.

Although DRC maintained a four year period (2001-2005) without any confirmed PV cases throughout the country (possibly due to implementation of immunization activities and especially those involving the national immunization days (NIDs) program), polio outbreaks occurred in the DRC in 2006 following importation from Angola [30]. WPV has virtually disappeared in DRC since 2011 [38], but efforts should be made towards eradication of poliomyelitis caused by neurovirulent VDPVs by maintaining high quality AFP surveillance, supplemented by environmental surveillance which is being initiated. These are essential for timely detection and response to potential WPV re-introduction and VDPVs re-emergence in DRC.

## Conclusion

The AFP surveillance system was efficient over the seven year period of 2008-2014 in the DRC with many indicators of performance above the minimum targets required by WHO. As a “gold standard” method for poliomyelitis detection, the continuous improvement of AFP surveillance is critical. Special efforts should focus on the timeliness of transportation of stool specimens to the polio reference laboratory. As long as WPV and circulating VDPVs strains are detected in any country worldwide, it will remain essential to achieve and maintain high quality AFP and environmental surveillance complying with required standards in order to ensure rapid detection and containment of PV circulation in DRC.

### What is known about this topic

- Only Afghanistan and Pakistan are the two remaining poliovirus-endemic countries worldwide and Africa has reported no wild poliovirus since the last case from Nigeria in July 2014;
- Coordinated and efficient responses to both wild poliovirus and vaccine-derived poliovirus related poliomyelitis outbreaks have led to the interruption of poliovirus infection in the Democratic Republic of Congo in 2011 (wild polioviruses) and 2012 (circulating vaccine-derived polioviruses).

### What this study adds

- Achievement and maintenance of high polio vaccine coverage remains essential for the prevention of poliovirus infection in the Democratic Republic of Congo;
- Despite the great results of the poliomyelitis surveillance and control, the timeliness of specimens’ transportation to the laboratory and non polio enterovirus isolation rate remains key surveillance indicators that require significant improvement to provide high confidence in the prevention of poliovirus infection in the Democratic Republic of Congo.
